# Detection of Microbiota during the Fermentation Process of Wine in Relation to the Biogenic Amine Content

**DOI:** 10.3390/foods11193061

**Published:** 2022-10-02

**Authors:** Ivana Regecová, Boris Semjon, Pavlina Jevinová, Peter Očenáš, Jana Výrostková, Lucia Šuľáková, Erika Nosková, Slavomír Marcinčák, Martin Bartkovský

**Affiliations:** 1 Department of Food Hygiene Technology and Safety, University of Veterinary Medicine and Pharmacy in Košice, Komenského 73, 041 81 Košice, Slovakia; 2 Department of Chemistry, Biochemistry and Biophysics, University of Veterinary Medicine and Pharmacy in Košice, Komenského 73, 041 81 Košice, Slovakia

**Keywords:** biogenic amines, mycobiota, must, PCR, terroir, wine, yeast

## Abstract

Viticulture is one of the traditional industries in Slovakia, where there are six wine-growing regions: Malokarpatska, Southern Slovakia, Central Slovakia, Nitra, Eastern Slovakia, and Tokaj. This study focuses on the detection of microbiota in soil samples, grape leaves and berries, and samples taken from fermenting must and young wine (the variety Tramín červený) in relation to the detected concentrations of biogenic amines during the fermentation process. In the examined samples, the number of yeasts and molds (from 3.8 to 6.8 log cfu/g or mL) and TVC (from 3.7 to 6.5 log cfu/g or mL) were determined via culture examination. At the same time, the number of LAB (from ˂3.0 to 4.4 log cfu/g or mL) was determined, which was the highest on day 4 of the must fermentation process and was related to the detected of the highest concentration of biogenic amines (histamine and tyramine) on day 6 in the investigated must samples using the UHPLC system. Mycobiota species were identified by MALDI-TOF MS, PCR, ITS-PCR-RFLP, and PCR sequencing of the amplified products. The study confirmed the presence of the yeasts *Saccharomyces cerevisiae, Metschnikowia pulcherrima, Hanseniospora uvarum, Pichia kudriavzevii, Pichia kluyveri, Pichia fermentas, Torulaspora delbrueckii, and Candida tenuis*. At the same time, the presence of molds (*Cladosporium herbarum, Cladosporium cladosporioides, Penicillium granulatum, Penicillium mononematosum, Botritis cinerea,* and *Penicillium glabrum*) was also confirmed in soil samples, leaves, grape berries, and fresh grape must. The study confirmed the reduction in the species diversity of the microbiota during the must fermentation process, which resulted in decreases in the concentrations of the monitored biogenic amines in the early stages of the must fermentation process and young wine of the variety Tramín červený.

## 1. Introduction

The Eastern Slovak wine-growing area is one of the most important wine-growing regions in Slovakia as well as in Europe, with an area of 1800 ha. It covers the territory of three regions, namely Zemplín, Abov, and Turňa. The vineyards contain heavy clayey to light sandy soils. The soil type is also influenced by the volcanic substrate of the Vihorlat Hills. Due to the volcanic origin, a higher minerality of the soil is ensured, which becomes part of the grapes and passes into the wine [1].

The various physical and chemical parameters of this environment determine the growth of plants in this geographical area (e.g., the temperature, humidity, precipitation, nutrient content in the soil, and sunlight). These natural factors have a significant impact on the occurrence of microorganisms in the ecosystem. Of all the microorganisms present on the surface of grapes, yeast is the most important [2]. Yeast populations on immature berries are low. At this stage, species such as *Rhodotorula*, *Cryptococcus,* and *Candida* occur in particular. Representatives of the genera *Saccharomyces*, *Hanseniaspora*, *Metschnikowia,* and *Zygosaccharomyces* predominate during the maturity stage [3]. The presence of yeast depends on local and climatic influences, altitude, grape variety, disease, and the degree of damage to the grapes. In general, the ripening of the grapes also increases the number of yeasts one to two times closer to the stem of the vine [4].

In addition to yeast, grapes, musts, and wines contain a number of bacteria that are more demanding in regards to environmental influences compared to yeast. In viticulture, lactic acid bacteria (LAB) are most often detected [5]. LAB are the major microorganisms responsible for the production of histamine in wine [6]. In addition to LAB, Tristezza et al. [7] showed that some yeasts other than *Saccharomyces* sp. (of wine origin) are also able to produce biogenic amines (BA).

These compounds are commonly found in different fermented foods (e.g., wine, beer, cheese, and sausages) [8,9]. The BAs in wine may have two principal sources: raw materials and fermentation processes. The presence of some amines in grapes (e.g., histamine and tyramine) has been previously reported [10]. These are mainly produced through enzymatic reactions such as decarboxylation, transamination, reductive amination, and the degradation of precursor amino compounds [11]. It is generally agreed that their concentration results are lower at the end of alcoholic fermentation (AF) and increase during malolactic fermentation [12]. However, even if less significant, BA formation by yeast during AF represents another common process [13]. Some factors (e.g., the winemaking process, storage conditions, quality related to raw materials, and potential microbial contamination) may affect the variability in BA content during winery operations [14,15,16,17]. Several studies have focused on the influence of other factors on the content of BAs in wine, such as grape variety [18], among others.

This study therefore focused on the detection of the concentration of the most common bioorganic amines in the must and young wine of the variety Tramín červený in relation to the population dynamics of microbiota in the process of primary must fermentation.

## 2. Materials and Methods

The examined samples were taken from the soil, berries, and vine leaves and from Tramin red must. Samples were taken from the Eastern Slovak wine-growing region, the Sobranecký region, and in the wine-growing village of Orechová. The samples were taken in September 2021. The must sample with a sugar content of 21.5 °Brix was poured into a 100 L stainless-steel container. Subsequently, it was spontaneously fermented, and samples were taken from the fermenting must on days 0, 2, 4, 6, and 8 and after 4 weeks of fermentation for an analysis of the presence of biogenic amines and the mycobiota of the fermenting must and young wine.

### 2.1. Physicochemical Measurements of Must during the Fermentation Process

Fermentation took place in stainless-steel tanks (wall thickness 0.5 mm, AISI 304 stainless steel). The temperature of the fermentation room was regulated at 18.0 ±0.1 °C. The fermentation temperature was not controlled. Fermentation took place for 4 weeks. For sugar content determination, two methods were used at the beginning of fermentation and during fermentation. The first method was the use of a Mettler-Toledo MyBrix handheld refractometer (Mettler-Toledo, Greifensee, Switzerland,) with a scale of 0–95 °Brix, and for control we used a modified titration method for the determination of sugars according to Rebelein method [19]. pH was measured potentiometrically using an electrode with a Hach Lange Pocket Pro + tester (Hach company, Loveland, Colorado, United States). The titratable acidity was measured using a Titra EVO Acid Automatic titrator (Dujardin-Salleron; Noizay, France). This method is based on the neutralization of the acids in must and wine samples to a neutral value of pH 7. At the same time, the titrator also determined the temperature of the sample. Alcohol was determined using an electric ebulliometer with electronic probe (Dujardin-Salleron; Noizay, France).

### 2.2. Culture Microbiological Examination

A stock suspension and further 10-fold dilutions were prepared from sterile 1 mL samples according to the instructions of ISO standard 6887-1 [20]. From the prepared dilutions, a microbiological culture examination of the samples was subsequently performed.

#### 2.2.1. Total Viable Count (TVC)

A 1.0 mL sample was taken from each respective dilution and inoculated in parallel into labeled, sterile petri dishes. The diluted samples were then mixed with 18 ± 2 mL of PCA agar broth cooled to 44–47 °C. After solidification, the inoculated broths were incubated in a thermostat at 30 ± 1 °C for 72 h. After cultivation, colonies were counted on the surface and inside the inoculated petri dishes. The plates containing between 10 and 300 colonies were used to calculate the number of microorganisms. The results were then converted to represent the amount in 1 mL of the sample according to ISO standard 4833-1 [21].

#### 2.2.2. Determination of Lactic Acid Bacteria (LAB)

Bacteria were isolated from the collected and examined samples according to ISO standard 6887-1 [20]. From three successive dilutions, 0.1 mL of the examined sample of De Man, Rogosa, and Sharpe agar selective diagnostic medium (Hi-Media, Mumbai, India) was inoculated as a smear. These samples were prepared and evaluated in parallel. Subsequently, the samples were incubated under anaerobic conditions to multiply the mesophilic lactic acid bacteria. The inoculated plates were incubated at 37 °C for 48 h using an AnaeroGen bag (Oxoid, UK). Furthermore, Petri dishes with colony counts between 10 and 150 were taken into account to determine the number of LABs.

#### 2.2.3. Determination of the Number of Yeasts and Molds

The quantitative determination was performed according to ISO standard 21527-1 [22]. The first three decimal dilutions were inoculated in a volume of 0.1 mL on the surface of Dichloran Rose-Bengal Chloramphenicol (DRBC) agar (Hi-Media, Mumbai, India) containing peptone, dextrose (glucose), potassium dihydrogen phosphate, magnesium sulphate, Rose Bengal, chloramphenicol, dichloran, and agar, with a final pH of 5.6 ± 0.2. The plates were then incubated at 25 °C for 5 (for yeast) or 8–10 days (for molds). After incubation, only inoculated petri dishes containing colony counts of less than 150 were selected for quantification. Subsequently, five colonies from the group with the same phenotypic characteristics were isolated from the surface of the agar medium and were used for further analysis.

### 2.3. Identification of Yeasts and Molds

Yeast and mold species identification was first performed by MALDI-TOF mass spectrometry (MS) by the comparison of the PMF of an unknown organism with the PMFs in the database according to the standard Bruker Daltonics [23] sample preparation protocol using formic acid and acetonitrile. The results were analyzed with an Ultra-flex III instrument (Bruker, Billerica, MA, USA) using Flex Analysis software (version 3.0) and were evaluated using BioTyper software (version 1.1) (Bruker, Billerica, MA, USA). After evaluation, species identification was performed using the following methods.

The total genomic DNA of the tested isolates was isolated by a modified method according to Regecova et al. [24] using zirconium and glass beads, Proteinase K (Macherey-Nagel GmbH & Co., Düren, Germany), ultrasonic waves, and the commercially available E.Z.N.A.^®^ Fungal DNA Mini Kit (OMEGA bio-tek, Norcross, GA, USA). DNA purity and concentration were measured using a BioSpec spectrophotometer (SHIMADZU, Korneuburg, Austria).

The rRNA region of the gene was amplified in a Thermal Cycler (Techne, Cambridge, UK). The primers used in the PCR reaction to amplify the ITS region (ITS1 and ITS4) were synthesized and used according to White et al. [25]. The PCR reaction proceeded as follows: initial denaturation at 95 °C for 5 min; 30 cycles of denaturation at 95 °C for 1 min, annealing at 53 °C for 2 min, and extension at 72 °C for 2 min; and final extension performed at 72 °C for 10 min. The obtained PCR products were digested by the restriction endonucleases *Mse*I, *Hha*I, *Hae*III, and *Hinf*I (New England BioLabs^®^inc., Ipswich, MA, USA). PCR products and restriction fragments were visualized via UV transillumination using a Mini Bis Pro^®^ (DNR Bio-Imaging Systems Ltd., Neve Yamin, Israel). Products with concentrations less than 40 ng did not appear on the electropherogram. The sizes of the individual fragments were detected using the GelAnalyzer 19.1 program. (version 14.0.0.0; Oracle Corporation, Santa Clara, CA, USA). The restriction profiles of the yeast reference strains (*Candida tenuis* ATCC 10,573 ™, *Pichia kluyveri* ATCC 9768 ™, *Pichia fermentas* ATCC 204,298 ™, *Metschnikowia pulcherrima* ATCC 52,710 ™, *Hanseniospora uvarum* ATCC 32,856 ™, *Torulaspora delbrueckii* ATCC 204289™, and *Saccharomyces cerevisiae* 9763™) were used to verify the correct yeast species, whereby identification took place via the ITS-PCR-RFLP method.

Identification by MALDI-TOF MS and ITS-PCR-RFLP using the endonucleases *Mse*I, *Hha*I, *Hae*III, and *Hinf*I was not sufficiently differentiated. Therefore, the identification of molds was performed using the conventional PCR method according to White et al. [25] described above (Section 2.3), and a subsequent sequencing of the obtained PCR fragments was performed by a commercial company (SEQme s.r.o., Dobříš, Czech Republic). The obtained isolates were sent to the GenBank—EMBL database for comparison with the sequences available in the nucleotide database of the National Center for Biotechnology Information (NCBI) available at http://www.ncbi.nlm.nih.gov/BLAST (accessed on 10 April 2022).

### 2.4. Determination of Biogenic Amines

The determination of biogenic amines in must and young wine was performed using ultra high performance liquid chromatography with a fluorescence detector. To analyze biogenic amines (histamine and tyramine) a Thermo Scientific UHPLC system (Dionex UltiMate 3000 RS) coupled with a fluorescence detector (FLD) was used. A YMC-Triart PFP column (150 × 3.0 mm, 1.9 μm) was used to separate the biogenic amines in the wine samples at a flow rate of 0.4 mL/min. The mobile phase consisted of (A) acetonitrile and (B) 0.1 mol/L ammonium acetate, and an isocratic elution of 55% (A): 45% (B) was applied. The column temperature was maintained at 25 ± 0.5 °C. A standard stock solution of investigated biogenic amines (BAs, histamine and tyramine) was prepared by dissolving each standard into deionized water to a concentration of 1000 mg/L. In the case of wine processing, the samples were initially diluted with hydrochloric acid (0.1 mol/L) at a ratio of 1:1 and then evaporated to dryness. Afterward, the aliquots of these biogenic amine standard stock solutions (at a concentration of 100 mg/L/sample residue) were added to 2 mL of deionized water. To the prepared solutions, 0.3 g of NaHCO3 and a dansyl chloride solution (2 mg/mL) were added. After sealing and mixing, the derivatization reaction was performed at room temperature for 90 min in the dark. Subsequently, toluene (4 mL) was added, to which the derivatized BAs were extracted. One milliliter of toluene extract was taken and evaporated to dryness. The residue was dissolved in 1 mL of acetonitrile and filtered through a 0.2 μm nylon syringe filter. The filtered solutions were used for UHPLC analysis in the amount of 5 μL. The excitation and emission wavelengths were set at 320 nm and 523 nm in the FLD detector, respectively. The identification of biogenic amines was carried out by comparing their retention time with their corresponding standard. The quantitation of biogenic amines in wine samples was performed by a standard curve generated by the ratio of the peak area to the concentration of each biogenic amine.

## 3. Results and Discussion

### 3.1. Quantitative Determination of Microorganisms

**Figure 1:**
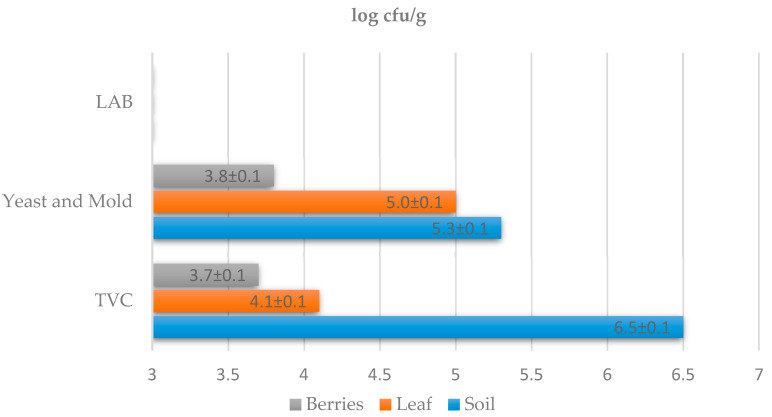
Quantitative microbiological culture examination of must and wine soil samples. TVC—total viable count; LAB—count of lactic acid bacteria; Yeast and Mold—count of yeasts and molds. The detection limit was ˂3.0 ± 0.0 log cfu/g.

Parameters such as the quantification of yeasts, lactic acid bacteria, and the total number of microorganisms (TVC) were monitored during the microbiological examination of the samples. Samples were taken from the must before fermentation (day 0), on days 2, 4, 6, and 8, and after 4 weeks of fermentation. The microbiological composition of the must is greatly influenced by the terroir. Regionally different characteristics of wine (terroir) are an important aspect of wine production. Grape and wine mycobiota represent regionally defined patterns associated with vineyards and climatic conditions [26]. Individual microbiological parameters change during fermentation. These parameters affect the final quality of the wine. In this study (Figure 1), the total number of microorganisms was determined on PCA agar, which ranged from 3.7 ± 0.1 to 6.5 ± 0.1 log cfu/g. The highest proportion of microorganisms was observed in the soil. Lactic acid bacteria in the must were detected in the samples in low numbers. The numbers of lactic acid bacteria found in all samples were ˂3.0 ± 0.0 log cfu/g. The numbers of yeasts in each sample ranged from 3.8 ± 0.1 to 5.3 ± 0.1 log cfu/g. Higher numbers of yeasts were found in the soil samples, at 5.3 ± 0.1 log cfu/g, followed by samples of vine leaves, with a representation of 5.0 ± 0.1 log cfu/g. The lowest proportion of yeasts was found on grape berries, at 3.7 ± 0.1 log cfu/g. The yeasts present are responsible for starting the fermentation process in the must, but after the multiplication of typical wine yeasts, their number gradually decreases.

**Figure 2:**
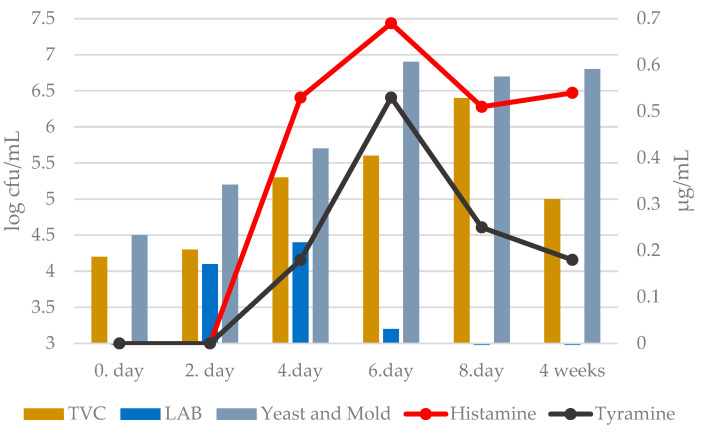
Quantitative microbiological culture examination in relation to the concentration of biogenic amines of must and young wine. TVC—total viable count (sd: ±0.1); LAB—count of lactic acid bacteria (sd: ±0.0); Yeast and Mold—count of yeasts and molds (sd: ±0.1).

Subsequently, in the quantitative microbiological culture examination of must and young wine samples, the total number of microorganisms ranged from 4.2 ± 0.1 to 6.4 ± 0.1 log cfu/mL (Figure 2). The lowest number was recorded on the 0th day after pressing the grape juice. Their numbers gradually increased and peaked on the 8th day after pressing (during the fermentation stage). After 4 weeks, the total number of microorganisms in the young wine decreased. The reduction in TVC in the young wine down to 5.0 ± 0.1 log cfu/mL was due to the presence of a higher alcohol content (Table 1).

The number of lactic acid bacteria in each must and young wine sample ranged from ˂3.0 ± 0.0 to 4.4 ± 0.1 log cfu/mL (Figure 2). We recorded the highest numbers of LAB on the second and fourth day after pressing the grape must. Similar values were also found by Kačáníová et al. [27], where LAB values in grape berries and fermented must samples ranged from 2.48 ± 0.1 to 4.52 ± 0.1 log cfu/g or mL.

The main lactic acid bacteria (LAB) isolated from wine are Lactiplantibacillus, Leuconostoc, Oenococcus, and Pediococcus genera [28,29], which positively influence wine by carrying out malolactic fermentation [30]. This process can increase wine aroma and mouthfeel, improve microbial stability, and reduce the acidity of wine. A growing number of studies support the appreciation that LAB can also significantly, positively and negatively, contribute to the sensorial profile of wine through many different enzymatic pathways. This is achieved either through the synthesis of compounds such as diacetyl and esters or by liberating bound aroma compounds such as glycoside-bound primary aromas and volatile thiols, which are odorless in their bound form. LAB can also liberate hydroxycinnamic acids from their tartaric esters and have the potential to break down anthocyanin glucosides, thus impacting wine color. LAB can also produce enzymes with the potential to help in the winemaking process and contribute to stabilizing the final product. For example, LAB exhibit peptidolytic and proteolytic activity that could break down the proteins causing wine haze, potentially reducing the need for bentonite addition. Other potential contributions include pectinolytic activity, which could aid juice clarification and the ability to break down acetaldehyde, even when bound to SO2, reducing the need for SO2 additions during winemaking [29].

With advanced fermentation, their numbers gradually decreased, which correlates with the measured value of the content of titratable acids after alcoholic fermentation, which was 7.89 at 28 days (Table 1). The values were lower than in the other measurements.

The number of yeasts and molds in each sample ranged from 4.5 ± 0.1 to 6.9 ± 0.1 log cfu/mL. The lowest numbers were recorded in samples of freshly squeezed grape must (day 0), with 4.5 ± 0.1 log cfu/mL. During the fermentation of the must, the numbers increased. The highest numbers were recorded during the harvest on the 6th and 8th days of fermentation, which were caused by so-called “stormy fermentation”. Saccharomyces yeasts are the chief microorganisms responsible for alcoholic fermentation in the wine industry. However, there is a growing interest in using non-Saccharomyces yeasts (Torulaspora delbrueckii, Lachancea thermotolerans, Metschnikowia pulcherrima, Schizosaccharomyces pombe, and Pichia kluyveri) [31]. These yeasts have weak fermentation capacity, but they play significant roles in wine quality, especially the aroma profile of wines. One of the effects of non-Saccharomyces yeasts on wine characteristics is due to their enzymes, which release substrates required for metabolic activities. The other effect is the formation of compounds such as volatile fatty acids and higher alcohols as a result of non-Saccharomyces yeast metabolism [32].

### 3.2. Identification of Mycobiota via MALDI-TOF Mass Spectrometry

After the quantitative microbiological culture examination of the samples, a more detailed detection of mycobiota was performed. Individual isolates were harvested from the surface of DRBC agar media according to macroscopic features such as colony staining, growth patterns, and colony shape. Five yeast colonies from a group with the same phenotypic characteristics and all mold colonies were isolated. Thus, 55 yeast isolates and 11 mold isolates were obtained. The initial identification was performed by MALDI-TOF mass spectrometry. This identification represented a rapid initial screening of the investigated mycobiota. As shown in Table 2, this method identified individual yeast species, where the scores of the identified isolates ranged from 1.789 to 2.330. For some isolates, the scores were below 1.700, indicating unreliable identification. As some results of yeast and mold isolate identification were not reliable or not identified according to the obtained score, isolate identification was performed using the ITS-PCR-RFLP method.

### 3.3. Identification of Mycobiota Using Other Molecular Methods

**Figure 3:**
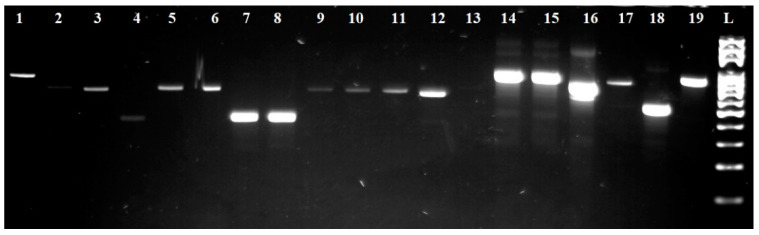
Electropherogram of a PCR reaction using ITS1/ITS4 primers. L: 100 bp ladder; lines 1, 14, 15, and 19: isolates *Saccharomyces cerevisiae*; lines 2 and 16: isolates *Hanseniospora uvarum*; lines 3, 5, 6, and 9–12: isolates *Candida tenuis*; line 4: isolates *Metschnikowia pulcherrima*; lines 7, 8, and 18: isolates *Pichia* sp.; line 17: isolates *Torulaspora delbrueckii*.

**Figure 4:**
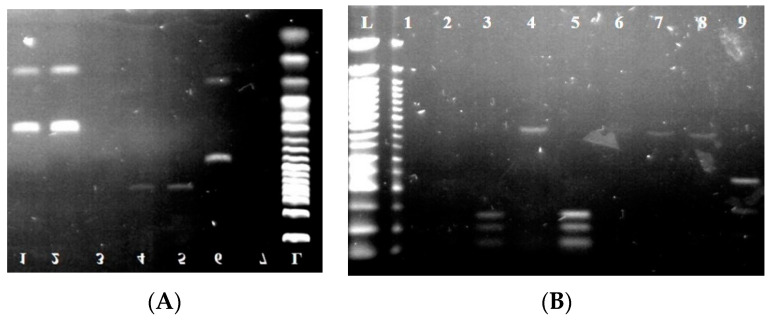

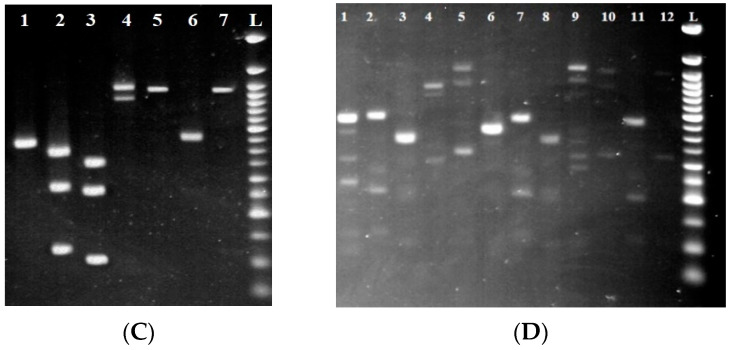
Electropherogram after the digestion of PCR products by restriction endonucleases. (**A**) (*Hae*III) L: 50 bp ladder; lines 1–5: isolates *Metschnikowia pulcherrima*; lines 4 and 5: isolates *Hanseniospora uvarum*; line 6: isolates *Candida tenuis*. (**B**) (*Hha*I) L: 50 bp ladder; lines 3 and 5: isolates *Pichia kluyveri*; line 9: *Torulaspora delbrueckii*. (**C**) (*Mse*I) L: 50 bp ladder; lines 2 and 6: isolates *Pichia kudriavzevii*; line 3: isolates *Pichia fermentas*. (**D**) (*Hinf*I) L: 50 bp ladder; line 1: isolates *Pichia fermentas*; lines 2 and 11: isolates *Metschnikowia pulcherrima*; lines 3, 8, 10, and 12: isolates *Saccharomyces cerevisiae*.

For further species identification, ITS-PCR-RFLP was performed using endonucleases *Hae*III, *Hinf*I, *Mse*I, and *Hha*I. The individual sizes of the PCR products and restriction fragments are listed in Table 3. At the same time, the visualization of the digested fragments is shown in Figure 3 and Figure 4.

Based on the results of the identification by ITS-PCR-RFLP, we were able to retrospectively confirm the accuracy of the identification via MALDI-TOF mass spectrometry. Eight species of yeast were identified: *Candida tenuis, Pichia kluyveri, Pichia fermentas, Pichia kudriavzevii, Metschnikowia pulcherrima, Hanseniospora uvarum, Torulaspora delbrueckii,* and *Saccharomyces cerevisiae*.

The *Hha*I endonuclease had the greatest resolution in yeast, which cleaved the obtained PCR products into fragments that were well-differentiated in the individual yeast species. The endonucleases *Hae*III and *Hinf*I had a lower resolution. Therefore, the endonuclease *Mse*I was used for more accurate identification. However, this endonuclease was not used in the case of *Saccharomyces cerevisiae* and *Torulaspora delbrueckii* due to its low resolution in these yeast species.

Mold isolates formed nonspecific fragments upon the cleavage of their PCR fragments by the above-mentioned endonucleases (as seen in Figure 4A–D). Therefore, the sequencing of the amplified PCR products was performed to identify molds. As seen from the results in Table 4, the largest number of isolates was isolated from soil samples (four isolates) and leaf samples (four isolates). At the same time, molds were still present in leaf samples, vine berries, and freshly pressed grape must. Molds were not detected in the samples of fermenting must and young wine.

### 3.4. Percentages of Mycobiota

The percentages of individual yeast species after prior identification were recalculated retrospectively based on phenotypic expression and colony growth on inoculated plates (Table 5). In the soil, the most represented species included Metschnikowia pulcherrima (7%), Candida tenuis (7%), and Pichia kluyveri (5%). Metschnikowia pulcherrima (4%) and Hanseniospora uvarum (4%) were most frequently detected in leaf samples. Metschnikowia pulcherrima (17%) and Hanseniospora uvarum (7%) were the most represented on grape berries. Felsöciová [4] also observed the presence of yeasts species on the surface of berries, including the genera Hanseniospora uvarum, Metschnikowia pulcherrima, Candida stellata, and small numbers of Saccharomyces sp.

The beginning of spontaneous fermentation was ensured by the species *Metschnikowia pulcherrima*, *Hanseniospora uvarum,* and *Pichia kluyveri,* which were mainly detected in the initial stages of fermentation. The numbers of these yeasts in freshly squeezed grape must were 27%, 15%, and 15%, respectively. These yeast species, however, were no longer present during the last harvest. Their percentages in the fermentation process gradually decreased with the onset of *Saccharomyces cerevisiae,* which is the main wine yeast. Its highest proportion was in the sample of young wine, with a percentage of 99%, while at the beginning of the fermentation process it represented only 10% of all yeast types. Its dominance indicates that the must is in the phase of turbulent fermentation (8th day of fermentation). The multiplication of other yeast species was suppressed in the later stages of fermentation.

Morata et al. [33] found that *Metschnikowia pulcherrima* are to some extent effective in reducing the ethanol content of wine. This is related to their aerobic respiratory metabolism, which under suitable aeration conditions by farmers, can aerobically metabolize more than 40% of sugars, thus significantly reducing the ethanol content. Ethanol production in wine is based on the ability of yeast strains to catabolize six-carbon molecules present in must into ethanol, a two-carbon compound [34]. The bioprocesses where yeasts convert glucose to ethanol are known as the glycolytic pathway followed by ethanol fermentation [35]. Fermentation (viz. the “anaerobic”-type transformation of glucose into ethanol) may occur despite the presence of O2 in the culture medium in significant concentrations when the initial concentration of the employed carbohydrate (i.e., glucose and/or fructose and/or sucrose) is higher than a “critical” value [36]. In fact, for a remarkable number of yeast species (the so-called “conventional” yeasts), even with a significant presence of oxygen in the fermentation medium (i.e., DOC values ≥ 20% *v/v* and in some cases >50% *v/v*), if the sugar concentration is higher than a critical (and in many instances not very high) concentration (e.g., ca 9 g/L or even lower), respiration is impossible; furthermore, despite the imposed oxygen saturation conditions, the microorganism shifts its metabolism completely towards the fermentative pathway and the subsequent biosynthesis and accumulation of ethanol into the medium [37].

The numbers of this type of yeast in our study continuously decreased during the fermentation process of must. In the last sampling of young wine, after 4 weeks of fermentation, only two species of yeast were identified: *Saccharomyces cerevisiae* at 99% and *Torulaspora delbrueckii* at 1%.

*S. cerevisiae* isolates play a crucial role in the quality of wine produced in defined regions. Isolates occurring in wine-growing environments have genetic variability adapted to the specifics of the ecosystem, which are reflected in the final product, as reported by Castillo et al. [38]. Viel et al. [39] examined the genetic diversity of the *Saccharomyces cerevisiae* population in the vineyards of three neighboring wine-growing areas with a protected designation of origin in north-eastern Italy. They found that in white grape varieties the presence of *Saccharomyces cerevisiae* in vineyards is very limited. On the contrary, it seems to be much more abundant in vineyards with blue must varieties. A greater occurrence of these yeasts was confirmed in the western part of the vineyard area. Furthermore, they found that industrial yeast isolates added prior to must fermentation suppress the original indigenous yeast isolates present in the vineyard must. The dominant yeast microflora in healthy grapes at harvest consists of species that survive in the must only during the first hours of fermentation. Other ascomycete genera, such as *Hanseniaspora, Candida, Pichia, Torulaspora, Kluyveromyces,* and *Metschnikowia* may survive longer and together dominate during the fermentation process until *Saccharomyces cerevisiae* takes over the alcoholic fermentation. In fact, these species far exceed the numbers of *Saccharomyces cerevisiae* on the surface of grape berries. After initial contact with grape sugars, they trigger alcoholic fermentation. However, according to Viel et al., *Saccharomyces cerevisiae* (initially in low numbers) uses its specific adaptive properties for rapid growth and becomes the main yeast species starting in the middle, turbulent fermentation phase [39]. In warmer and drier years, *Saccharomyces* sp. in the must are present in higher numbers than in the colder and wetter vintages [40]. *S. cerevisiae* is found in grapes in very low concentrations, while other yeast species are present in much larger populations in grapes and grape must. Nevertheless, it plays a dominant role during the fermentation process because it is able to survive the stressful conditions of fermentation and thus becomes the dominant yeast in the turbulent fermentation phase, during which non-*Saccharomyces* yeasts are unable to survive and multiply due to a higher alcohol concentration. At the same time, *S. cerevisiae* is primarily responsible for the formation of higher alcohols, esters, and aldehydes, which have the greatest influence on the fermentation bouquet. From a quantitative point of view, higher alcohols are the most important groups of compounds that *S. cerevisiae* produces during fermentation [40,41,42]. Kraková et al. [43] monitored yeast diversity in the samples from which the isolates were obtained, which came from various wine-growing regions of Slovakia, namely the Malokarpatska wine-growing region around Modra and the Southern Slovak region in the vicinity of Strekov. Before the fermentation of the Frankovka modrá grapes from Strekov, they obtained the yeast species *Candida zemplina* and *Metschnikowia* sp. with an incidence of 50%. Only one species of yeast, *Hanseniaspora uvarum* has been identified in Modra. *Pichia fermentans* and *Pichia kluyveri* represented up to 56.67% of the Veltlínske zelené in the Strekov variety. In addition, *Hanseniaspora uvarum* and *Metschnikowia* sp. represented 16.67%. The last genus, with a representation of 10%, was *Metschnikowia chrysoperlae*. In Modra, 56.67% of the species were *Hanseniaspora uvarum*, 33.33% of the species were *Pichia fermentans* and *Pichia kluyveri,* and 10% of the genera were *Metschnikowia* sp. [44,45]. It is the genera *Hanseniaspora, Candida, Kluyveromyces, Cryptococcus, Kloeckera, Pichia, Metschnikowia, Rhodotorula, Torulaspora,* and *Zygosaccharomyces Saccharomyces cerevisiae* that contribute significantly to the formation of wine aroma [46].

In addition to yeasts, our work also identified individual species of molds, which were detected only in soil samples, leaves, berries, and grape juice, namely *Cladosporium herbarum, Cladosporium cladosporioides, Penicillium granulatum, Penicillium mononematosum, Botritis cinerea,* and *Botritis cinerea*. According to Barata et al. [47] and Liu et al. [48], in freshly crushed grape must, mold communities are very diverse and characterized by genera such as *Aureobasidium, Cladosporium, Penicillium,* and *Botritis,* which come from a wine-growing ecosystem. However, geographical signatures decrease during the spontaneous fermentation of wine as the growth of yeast decreases the diversity and composition of the community, especially *S. cerevisiae*.

Yeast has the greatest influence on the sensory properties of wine. The aroma and taste of the wine are important characteristics that create the differences between the different types of wine [4].

### 3.5. Determination of Biogenic Amines in Must and Young Wine

To monitor the presence and changes in the concentrations of biogenic amines (histamine and tyramine) in the must during fermentation, we used samples of Tramin red of the year 2021. As shown in Figure 2, increase in the concentrations of both monitored BAs occurred during the same phase of the fermentation process. A sharp increase in the concentration of histamine in the must was recorded between the 2nd and 6th day of must fermentation. The tyramine concentration rose sharply between the 4th and 6th days. The highest concentrations of monitored BAs were recorded on day 6.

Yeast is involved in BA production during precisely the same time as LAB. There is a general agreement that yeast produces a less significant proportion than LAB in terms of the final BA content of wine. Several studies on yeast production have been performed, and most have only quantified histamine [13,49].

Torrea et al. [50] found moderate isolate production of *S. cerevisiae* BA, but concentrations were very low. Tristezza et al. [51] demonstrated the ability of yeast to produce histamine during grape must fermentation. They detected one isolate of *M. pulcherrima* that was able to synthesize histamine. In contrast to these authors, Landete et al. [52] did not detect BA production in any of the 36 isolates of the various wine yeast genera examined: *Aureobasidum, Candida, Hanseniaspora, Hansenula, Kloeckera, Metschnikowia, Pichia,* and *S. cerevisiae* isolates. These results lead to the conclusion that yeast does not appear to be the main producer of most amines found in wine. Usually, BA production results from the presence of bacteria that are capable of decarboxylating amino acids. For example, histamine is formed from histidine by histidine decarboxylase (hdc), and tyrosine is a precursor of tyramine produced by tyrosine decarboxylase (tdc) [10,53]. In our study, the highest concentrations of histamine and tyramine were found on the 6th day of must fermentation, which correlated with a higher number of LAB in the samples as well as with an increasing number of yeasts, especially *S. cerevisiae*. It is histamine and tyramine that are considered to be the biogenic amines found in Soucheos wines [54]. In general, amines play an important metabolic role in living cells. Polyamines are essential for growth; amines other than histamine and tyramine are involved in the function of the nervous system and blood pressure control. Biogenic amines are undesirable because, if absorbed excessive high concentrations, they can cause headaches, difficulty breathing, palpitations, hypertension or hypotension, and several allergic disorders [55]. The most toxic BA is histamine, and its effect may be potentiated by other amines [56].

## 4. Conclusions

The study confirmed the biodiversity of the mycobiota in the variety Tramin červený, originating from the Eastern Slovakia wine-growing region. It pointed out the close relationship between LAB and non-*Saccharomyces* yeasts and the production of histamine and tyramine. At the same time, it confirmed the weak correlation between the yeast *Saccharomyces cerevisiae* and the formation of the monitored BAs since, in the later stages of stormy fermentation, despite the detected high number of this type of yeast, there was a decrease in the concentrations of the investigated BAs.

## Figures and Tables

**Table 1:** Physicochemical measurements of must during the fermentation process.

Parameters	Day 0	1 Week	2 Weeks	3 Weeks	4 Weeks
sugar content (°Brix)	21.5	17.4	12.54	7.54	4.32
sugar content (g/L)	21.85	16.52	11.89	6.94	4.15
pH	3.21	3.18	3.24	3.48	3.79
titratable acidity (g/L^1^)	8.79	8.69	8.72	8.19	7.89
alcohol content (%)	0	2.12	5.48	8.78	11.42
temperature (°C)	18.54	18.81	18.73	18.62	18.10

**Table 2:** Identified yeast species from examined soil samples, leaves, berries, must, and new wine.

Species	MALDI TOF MS (Score Value)	ITS-PCR-RFLP
*Candida tenuis*	+ (2.000–2.060)	+
*Pichia kluyveri*	+ (1.870–2.016)	+
*Pichia fermentas*	+ (1.789–2.101)	+
*Pichia kudriavzevii*	+ (1.984–2.104)	+
*Metschnikowia pulcherrima*	+ (2.010–2.105)	+
*Hanseniospora uvarum*	+ (1.900–2.330)	+
*Torulaspora delbrueckii*	+ (1.791–2.140)	+
*Saccharomyces cerevisiae*	+ (1.890–2.180)	+

(+)—identified isolate.

**Table 3:** PCR products and restriction patterns of yeast.

Yeast Species	PCR Product	RFLP-ITS-PCR			
	bp	*HaeIII*	*HinfI*	*MseI*	*HhaI*
*Candida tenuis*	680	500; 150	300; 180	370; 120; 50	320; 130
*Pichia kluyveri*	470	375; 85	250; 220	271; 95;	141; 98; 69
*Pichia fermentas*	470	340; 85	260; 210	150; 120	170; 110; 80
*Pichia kudriavzevii*	470	361; 71; 31	198; 137; 135	466	185; 158; 69
*Metschnikowia pulcherrima*	450	285; 100	200	265; 52	210; 100
*Hanseniospora uvarum*	750	750	350; 200; 180	300; 140; 100	320; 105
*Torulaspora delbrueckii*	850	845	410; 380; 100	-	210; 130; 100
*Saccharomyces cerevisiae*	900	310	360	-	360; 340

**Table 4:** Identification of isolated molds from the examined samples.

Isolate Number	Species	Number of ITS Sequence in Gen Bank
PLM1	*Cladosporium herbarum*	MT524447.1
PLM2	*Botritis cinerea*	MT573470.1
PLM 3	*Cladosporium cladosporioides*	ON005144.1
PLM 4	*Cladosporium cladosporioides*	LR778221.1
PLM 5	*Botritis cinerea*	MK562062.1
PLM 6	*Botritis cinerea*	MT573470.1
PLM7	*Penicillium granulatum*	MT598824.1
PLM 8	*Cladosporium cladosporioides*	MT573472.1
PLM9	*Cladosporium cladosporioides*	MT466517.1
PLM10	*Penicillium mononematosum*	MN794476.1
PLM11	*Penicillium glabrum*	MT441616.1

**Table 5:** Percentages of individual yeast species.

Species Yeast	Soil	Leaf	Berries	Must and Young Wine					
				Day 0	Day 2	Day 4	Day 6	Day 8	4 Weeks
*Candida tenuis*	7%	1%	4%	11%	11%	10%	8%	1%	-
*Pichia kluyveri*	5%	1%	5%	15%	14%	14%	12%	-	-
*Pichia fermentas*	2%	1%	4%	8%	7%	5%	-	-	-
*Pichia kudriavzevii*	4%	3%	4%	7%	3%	-	-	-	-
*Metschnikowia pulcherrima*	7%	4%	17%	27%	26%	15%	9%	-	-
*Hanseniospora uvarum*	3%	4%	7%	15%	11%	3%	2%	-	-
*Torulaspora delbrueckii*	2%	2%	3%	7%	11%	16%	22%	5%	1%
*Saccharomyces cerevisiae*	5%	1%	4%	10%	17%	37%	47%	94%	99%

## Data Availability

Data is contained within the article.
